# Challenges and Insights in Managing Device-Related Thrombosis Post-WATCHMAN Implantation: A Case Report of Surgical Thrombectomy in an Elderly Jehovah’s Witness Patient With Atrial Fibrillation

**DOI:** 10.7759/cureus.49266

**Published:** 2023-11-22

**Authors:** Yusuke Tsukioka, Valluvan Jeevanandam, Blaine Johnson

**Affiliations:** 1 Cardiac/Thoracic/Vascular Surgery, University of Chicago Medicine, Chicago, USA

**Keywords:** jehovah's witness patient, surgical thrombectomy, atrial fibrillation (af), device-related thrombosis (drt), watchman device

## Abstract

The WATCHMAN device offers a viable alternative to long-term oral anticoagulation for stroke prevention in nonvalvular atrial fibrillation, particularly for high-risk patients. Despite its success, device-related thrombosis (DRT) remains a concern, potentially restricting its wider use. We present an 83-year-old female Jehovah's Witness with atrial fibrillation who, after successful WATCHMAN device implantation, suffered multiple transient ischemic attacks six months later. Initial investigation revealed a thrombus on a slightly exposed strut of the almost completely endothelialized device. Despite treatment with warfarin and rivaroxaban, urgent surgical intervention was ultimately required to remove the thrombus completely. This case illustrates the risk of thrombus formation even with minimal strut exposure and the challenges in managing DRT. It also highlights the necessity for diligent monitoring and potential reassessment of post-implantation anticoagulation protocols. Our report adds to the limited literature regarding surgical thrombectomy following WATCHMAN implantation and provides valuable insights for clinicians managing similar scenarios.

## Introduction

The WATCHMAN device (Boston Scientific Corporation, Marlborough, MA, USA) serves as an effective alternative to oral anticoagulants for thromboembolic prevention in nonvalvular atrial fibrillation, particularly in high-risk patients, with favorable outcomes in procedural success and ischemic event prevention [1,2]. However, significant challenges, such as the occurrence of device-related thrombosis (DRT), restrict the broader application of left atrial appendage closure (LAAC) to a wider patient population [3]. In this case report, we describe a patient who experienced multiple transient ischemic attacks (TIAs) presumed to be caused by embolization of a thrombus within the left atrium six months after WATCHMAN device placement. Although the WATCHMAN was almost entirely endothelialized, thrombus formation was observed on a minimally exposed stent strut, which was considered the source of thrombogenesis. This report elucidates the anatomical characteristics using clear images, including diagnostic imaging, the thrombus's nature, and the endothelium's state at the thrombus attachment site. No published literature exists of this nature. Therefore, this report has significant clinical implications and valuable insights for clinicians managing similar scenarios.

## Case presentation

History of presentation

**Figure 1:**
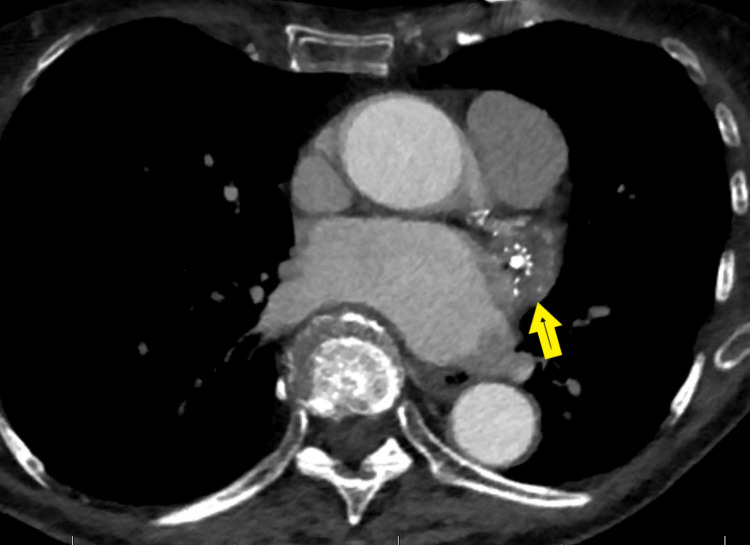
Watchman device in the left atrial appendage The yellow arrow indicates the location of the WATCHMAN device and associated clots within the left atrial appendage.

**Figure 2:**
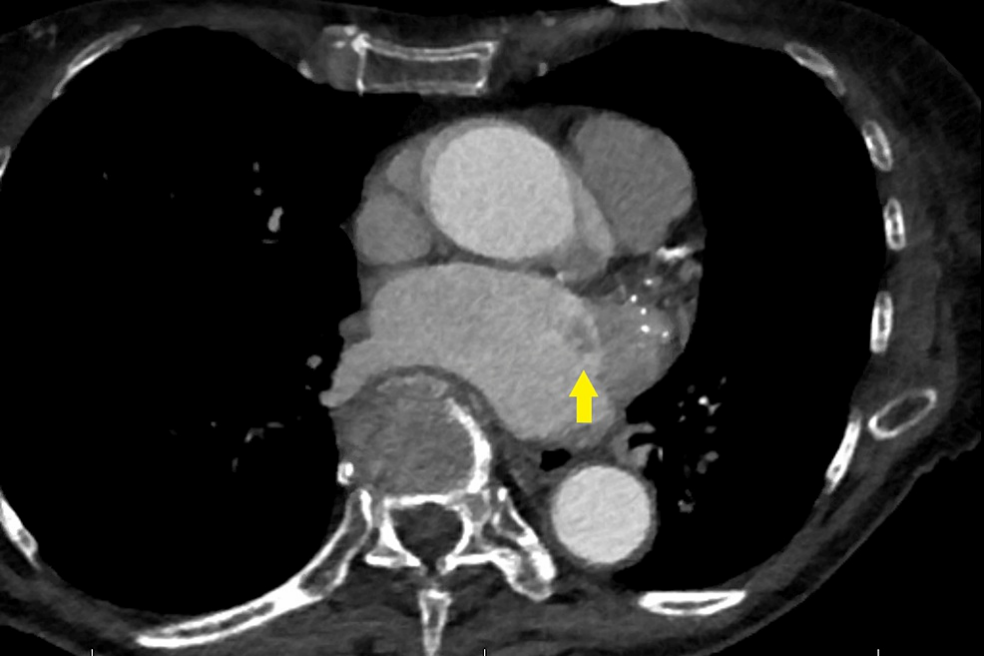
Thrombus in the left atrium. The yellow arrow points to a thrombus attached to the strut of the Watchman device within the left atrial appendage.

The patient, an 83-year-old female Jehovah's Witness with a height of 162 cm and a weight of 51.7 kg (Body Mass Index: 19.6 kg/m², Body Surface Area: 1.5 m²), presents with a past medical history that is significant for persistent atrial fibrillation. In 2019, a WATCHMAN 2.5 device was placed at an outside hospital, followed by approximately 45 days of postoperative Warfarin therapy. Details regarding her other prescribed medications are unknown. Postoperatively, her initial recovery was smooth until six months post-implantation, when she began experiencing repeated hospitalizations due to Transient Ischemic Attacks (TIAs). A computed tomography (CT) angiogram revealed a thrombus on the left atrial appendage side of the Watchman device, featuring a 9 mm stalk (as illustrated in Figures 1-2).

Consequently, she was started on rivaroxaban. Despite 18 months of rivaroxaban therapy, the thrombus persisted, although there were no recurrent TIA symptoms. This led to her referral to our institution for surgical thrombectomy.

The mitral valve leaflets are thickened and have a myxomatous appearance, with posterior leaflet prolapse leading to moderate mitral regurgitation and mild mitral annular calcification. The pressure gradient across the mitral valve was below 5 mmHg, indicating no significant mitral stenosis.

The patient's pre-operative laboratory results included a hemoglobin of 13.2 g/dL, platelet count of 184x10^9^/L, white blood cell count of 5.5 x 10^9^/L, creatinine of 0.66 mg/d, glomerular filtration rate of 83 mL/min/1.73m² and a Prothrombin Time and International Normalized Ratio (PT-INR) of 1.0. The CHA2DS2-VASc score was 5. The Society of Thoracic Surgeons Risk Score indicated a 3.81% risk of operative mortality. Due to her moderate mitral regurgitation, non-surgical thrombus aspiration was not selected as a treatment option.

Management

**Figure 3:**
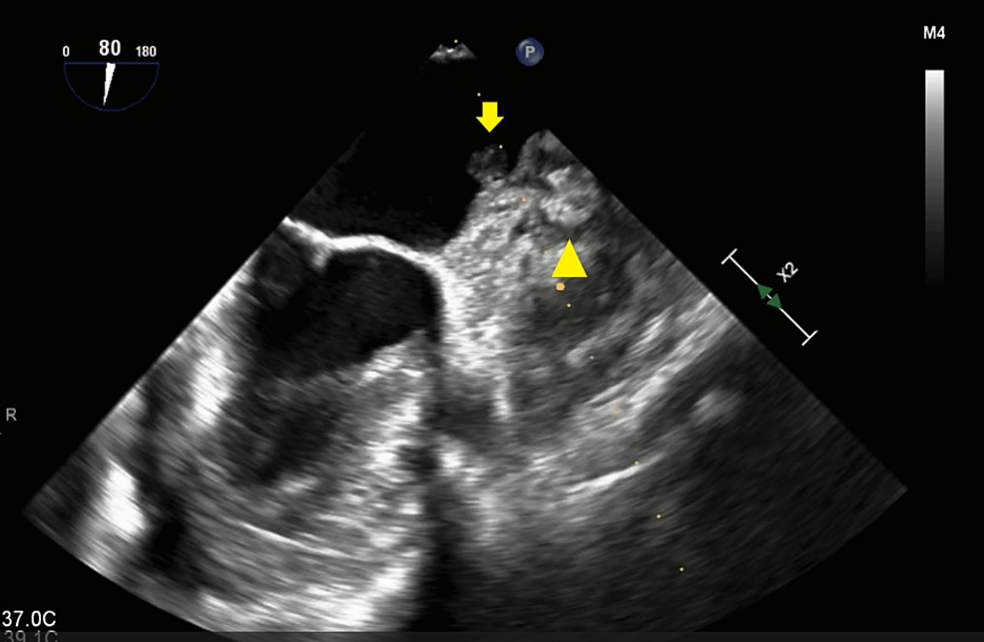
Thrombus in the left atrium and left atrial appendage. The yellow arrow indicates a thrombus in the left atrium. The yellow triangle indicates the thrombus and WATCHMEN device in the left atrial appendage.

**Figure 4:**
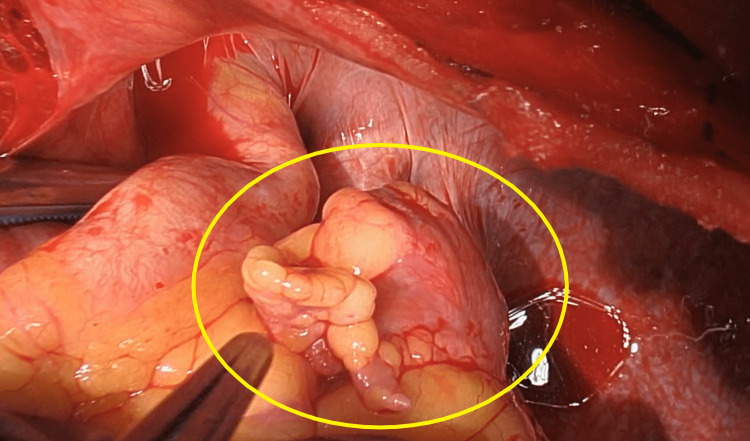
Left atrial appendage. The left atrial appendage was palpated and felt firm due to the Watchman device and clots within it.

Surgical intervention was completed under general anesthesia in the supine position. A routine median sternotomy was performed, and the adhesions were dissected. Cardiopulmonary bypass preparation included activated clotting time-guided heparinization. The ascending aorta was cannulated with a purse string suture and a 20 Fr aortic cannula. Bicaval cannulation included a 24 Fr dual lumen peripheral (DLP) right-angle metal venous cannula for the superior vena cava and a 30 Fr DLP straight venous cannula for the inferior vena cava. Before initiation of CPB, acute normovolemic hemodilution (ANH) is achieved via the sequestration of heparinized venous whole blood. The aorta was cross-clamped, and blood microplegia was administered via an antegrade cardioplegia cannula to induce cardiac arrest, followed by retrograde administration of cold maintenance every 20 minutes and warm straight blood before reperfusion. The patient was cooled to 34°C. Intraoperative transesophageal echocardiography (TEE) revealed a mobile 1 cm mass attached to the Watchman device (Figure 3). The left atrial appendage was palpated and felt firm due to the WATCHMEN device and clots within it (Figure 4).

**Figure 5:**
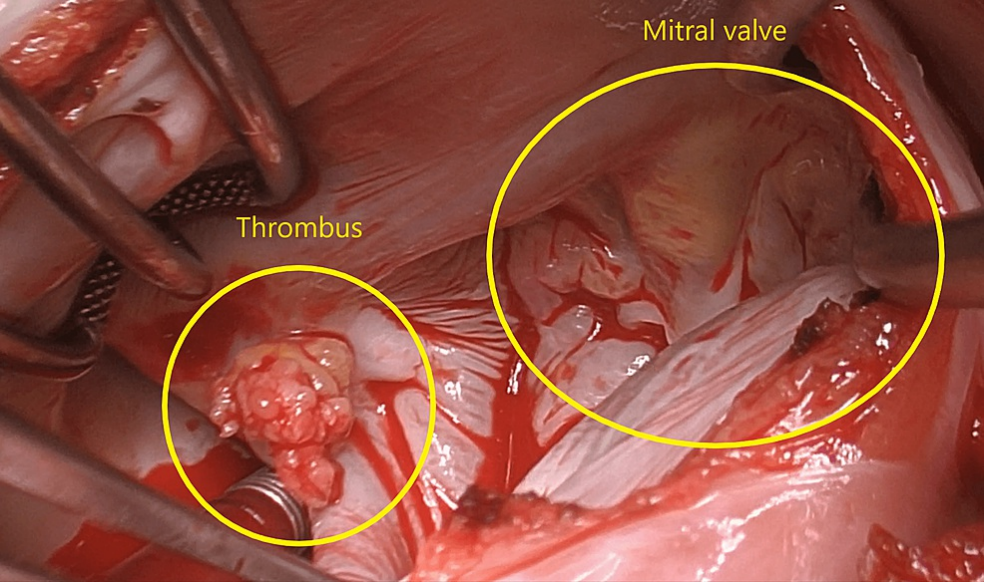
Thrombus and mitral valve. The left atrium was exposed using an extended septal approach through the right atrium, and the thrombus (measuring 1.1 x 0.5 x 0.3 cm) was found.

**Figure 6:**
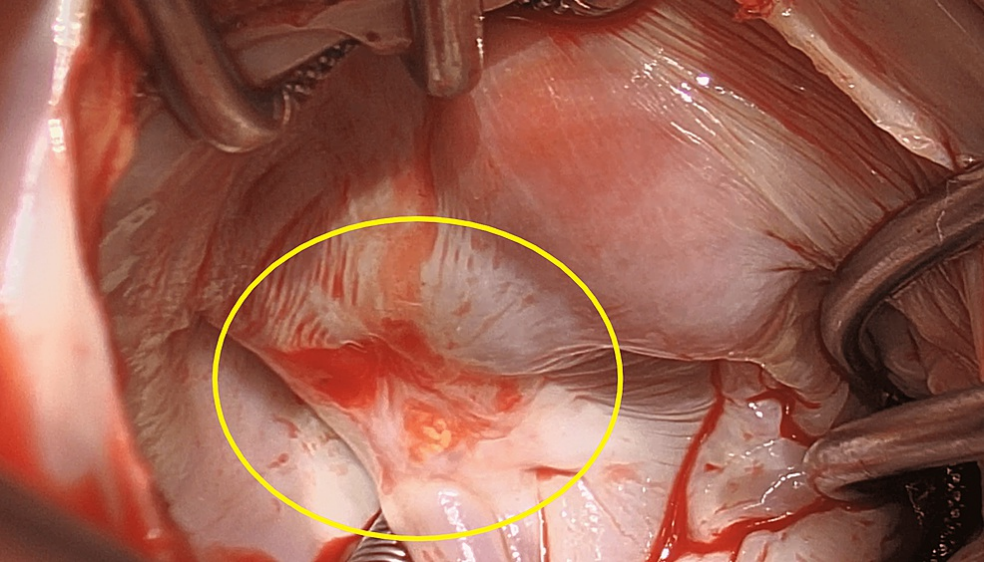
Endothelialized WATCHMAN device The Watchman device was well endothelialized, with the thrombus attached to one of its struts, which was minimally exposed.

**Figure 7:**
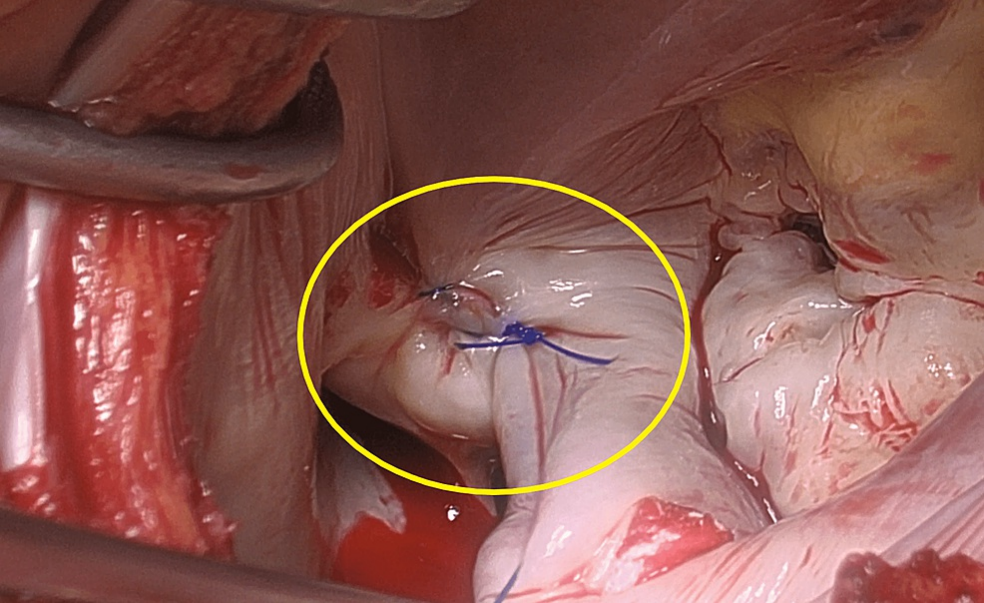
Stitch on minimally exposed WATCHMAN device The minimally exposed WATCHMAN device was covered with the endocardium of the left atrium using a 3-0 suture.

The left atrium was exposed using an extended septal approach through the right atrium, and the thrombus (measuring 1.1 x 0.5 x 0.3 cm) was excised (Figure 5). The specimen was sent for culture and pathological examination. The Watchman device was well endothelialized, with the thrombus attached to one of its struts, which was minimally exposed (Figure 6). The area was covered with the endocardium of the left atrium using a 3-0 suture (Figure 7).

The mitral valve leaflets exhibited myxomatous disease, with the P2 segment being too distorted for repair. A 33 mm St. Jude Epic Porcine Mitral valve (St. Jude Medical, Inc., St. Paul, MN, USA) was implanted using 18 pledgeted mattress 2-0 TiCron sutures (Medtronic, Minneapolis, MN, USA), with the pledgets placed on the atrial side. Efforts were made to preserve the maximal number of anterior and posterior chordae tendineae. The valve seating was confirmed to be excellent.

The patient was subsequently rewarmed and separated from CPB successfully. After complete heparin reversal, the sequestered ANH volume was transfused in a continuous circuit. The total CPB time was 106 minutes, with a cross-clamp time of 77 minutes and a reperfusion time of 17 minutes. The patient's DuBois body surface area was 1.54 m^2^, resulting in a calculated flow of 3.69 L/min at a 2.4 cardiac index.

The pathology report indicated that the excised mass was an organizing thrombus with significant acute inflammation. No bacterial growth was detected in the culture. Postoperative TTE showed normal left and right ventricular function and regular movement of mitral bioprosthesis leaflets. No thrombus was present in the left atrium. The patient's hemodynamics remained stable, and she was discharged home seven days after the operation with warfarin and aspirin. The patient's PT-INR levels have been consistently well-managed within the range of 2.0 to 3.0. A CT scan conducted two months after the surgery revealed no thrombus in the left atrium.

## Discussion

This case report highlights several challenges in managing Jehovah's Witness patients with atrial fibrillation who have undergone prior LAAC with the WATCHMAN device to reduce the risk of stroke. In this case, despite adequate anticoagulation therapy with warfarin, the patient experienced multiple TIAs believed to be caused by a thrombus in the left atrium that occurred approximately six months post-implantation. Anticoagulation with rivaroxaban was also unsuccessful, leading to the need for urgent surgical thrombectomy. Reports of surgical thrombectomy following WATCHMAN implantation are limited, making this case a valuable addition to the literature.

According to a meta-analysis by Song et al., the incidence of device-related thrombus (DRT) post-left atrial appendage closure (LAAC) is 2.8%, with a stroke incidence of 11.5% in the DRT group compared to 2.9% in the non-DRT group (OR: 5.08; 95% CI = 3.47-7.44), indicating an association between DRT and increased stroke risk. Zhu et al. demonstrated no significant difference in thromboembolic events between the Watchman 2.5 and the Amplatzer Amulet Left Atrial Appendage Closure Device (Abbott Laboratories, United States). Predictors of DRT include smoking, female sex, CHADS2 and CHA2DS2-VASc scores, platelet count, and ejection fraction (EF) [4-7].

The optimal anticoagulation management following WATCHMAN device implantation remains unclear. Warfarin and aspirin are the most commonly prescribed medications for the first 45 days, followed by six months of dual antiplatelet therapy (DAPT) from the day of the procedure, and then lifelong aspirin. For patients with absolute contraindications to oral anticoagulants, six months of DAPT post-device implantation is considered safe and effective, followed by lifelong aspirin [8]. In this case, while there is a lack of clarity regarding the patient's medication history post-Watchman device placement, it may be necessary to discuss whether antiplatelet therapy was administered following Warfarin treatment.

In this case, only a minimal portion of the Strut was exposed, with the rest being endothelialized. This suggests that even minimal exposure to the Strut can lead to thrombus formation. When performing surgical thrombectomy after WATCHMAN implantation, as in this case, it is crucial to cover and secure the exposed parts of the device with the endocardium to prevent thrombus formation.

The primary limitation of this approach is the risk involved with anesthesia and the bloodless operation for surgical thrombectomy. To that end, our patient was optimized preoperatively by the bloodless cardiac surgery service and managed intraoperatively by a cardiac anesthesia team.

This case also highlights the importance of a multidisciplinary approach to complex bloodless cardiac surgery. Coordination between interventional cardiology, cardiac surgery, cardiac anesthesia, and perfusion services within our heart and vascular center allowed urgent surgical intervention. In this setting, surgical thrombectomy for DRT may be an option for other patients presenting complications from WATCHMAN device deployment.

## Conclusions

This case report highlights the complexity of managing bloodless patients with the WATCHMAN device, particularly in DRT. Despite the device's high rate of endothelialization, our patient experienced TIAs due to a thrombus on a minimally exposed strut, necessitating surgical intervention. This case emphasizes the need for vigilant postoperative monitoring and may prompt reconsideration of anticoagulation strategies following WATCHMAN implantation. Our experience with surgical thrombectomy in this context is a novel contribution to the literature and could inform future clinical practice.
